# Acute Limb Ischemia Caused by Embolus of Primary Lung Cancer Complicating Trousseau’s Syndrome

**DOI:** 10.3400/avd.cr.21-00102

**Published:** 2022-03-25

**Authors:** Chiharu Tanaka, Fumio Sakamaki, Hidekazu Furuya, Masaomi Yamaguchi, Kazuo Kanabuchi, Kenji Kuwaki

**Affiliations:** 1 Department of Cardiovascular Surgery, Tokai University Hachioji Hospital, Hachioji, Tokyo, Japan; 2 Department of Respiratory Medicine, Tokai University Hachioji Hospital, Hachioji, Tokyo, Japan

**Keywords:** tumor embolism, acute ischemic limb, embolectomy

## Abstract

Limb ischemia caused by tumor embolus is rare. In this study, we report the case of a 77-year-old woman who suffered from acute ischemic limb. Computed tomography showed a tumor in the right bronchus invading the left atrium. The tumor fragments scattered resulting in the occlusion of the right iliac artery. The excluded embolus was revealed as a squamous cell carcinoma. Regarding the popliteal venous thrombus, Trousseau’s syndrome was complicated. The patient was discharged without any complications. We believe that advanced lung cancer is a differential diagnosis of acute ischemic limbs and that successful limb rescue contributed to a patient’s quality of life.

## Introduction

Tumor embolism associated with malignancy is extremely rare. In this study, we report a case of a patient with acute limb ischemia due to primary lung cancer, who underwent emergent embolectomy for limb salvage and diagnosis.

## Case Report

**Figure 1:**
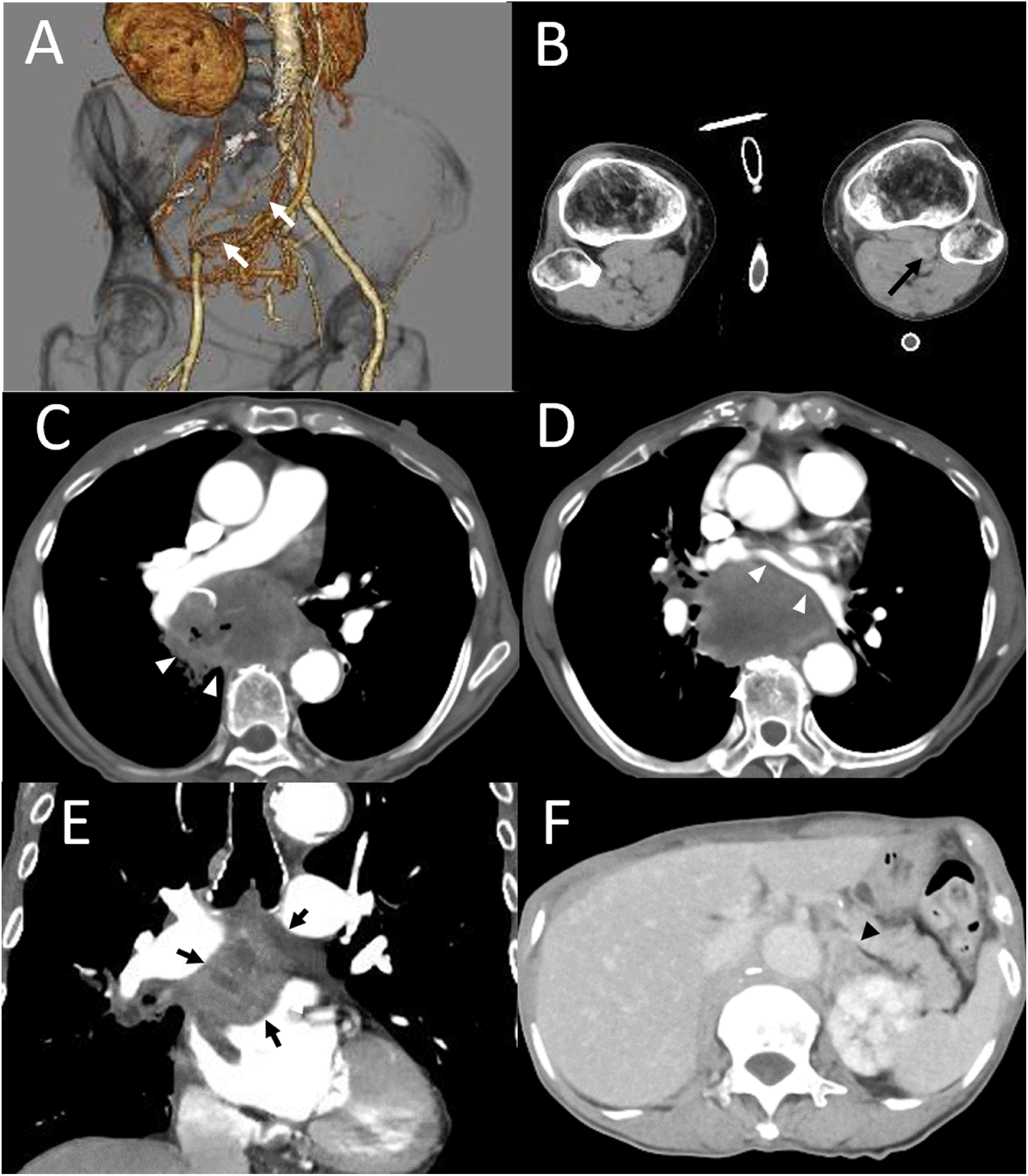
Fig. 1 Images of the computed tomografic scan. (**A**) Arrows indicate the occluded right common and external iliac arteries. The left iliac artery was patent. (**B**) The deep venous thrombus was observed in the left popliteal vein, as indicated by the arrow. (**C**) The tumor occupied and obstructed the right middle and lower lobar bronchi, as indicated by the arrowheads. (**D**) The tumor pressed the left atrium (LA) and left superior pulmonary vein, as pointed out by the arrowheads. (**E**) The arrows point to the mass in the LA. There was no clear distinction between the tumor invading the right main bronchus and the mass formed in the LA. (**F**) The left adrenal gland was swollen with a mass, as indicated by the arrowhead.

**Figure 2:**
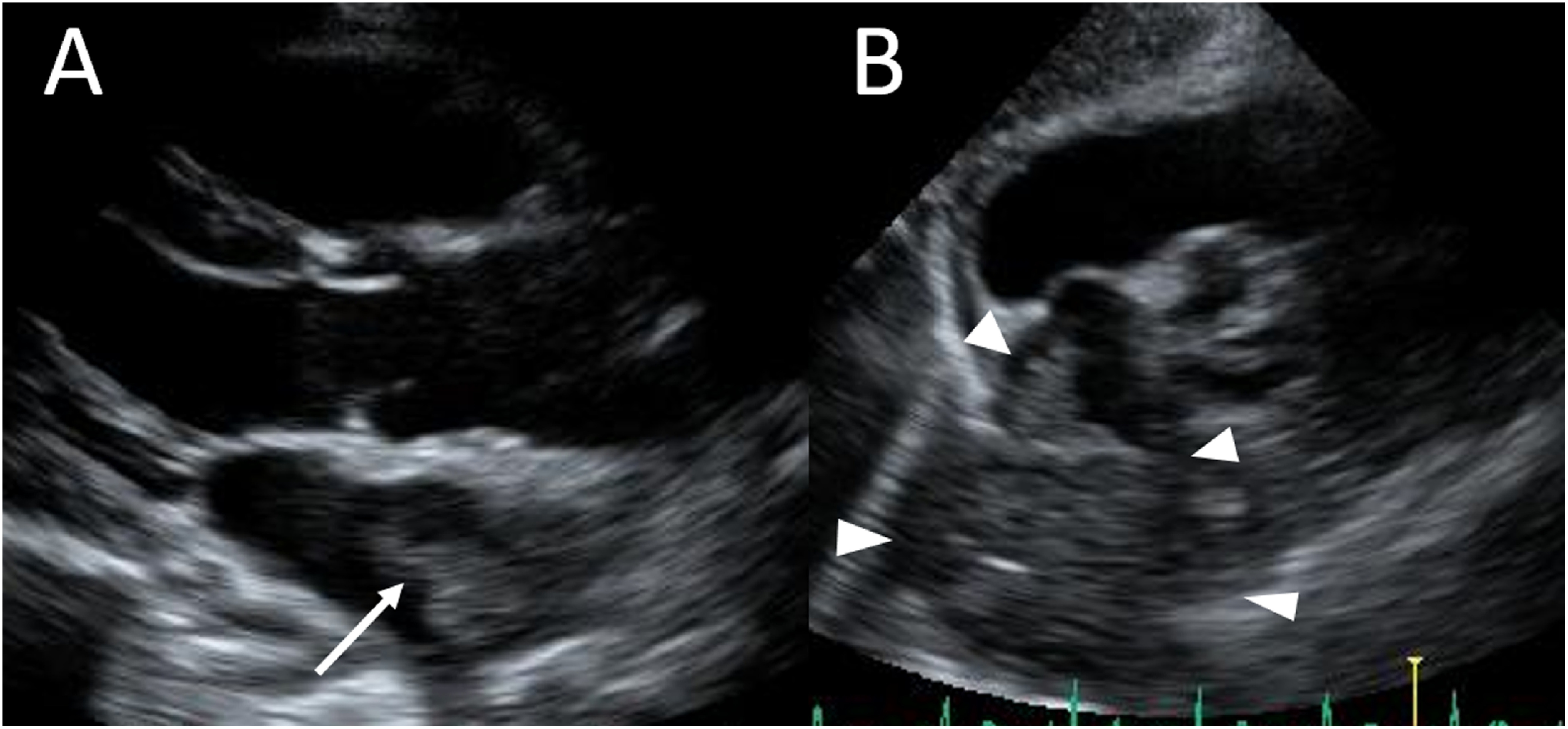
Fig. 2 Echocardiographic findings. (**A**) There was a highly mobile mass, as indicated by the arrow, in the left atrium (LA). (**B**) The mass in the LA had continuity with the tumor in the right main bronchus, as suggested by the arrowheads.

A 77-year-old woman presented to the emergency department with sudden onset of pain, cold sensation below the right knee, and dyspnea while riding a bicycle. A history of hypertension that was controlled with tablets was noted. She was an active smoker, smoking 20 cigarettes per day, and presented with a loss of appetite for the past 2 months. She was conscious, and her blood pressure was 180/70 mmHg, heart rate was 98 beats per minute, respiration rate was 24 breaths per minute, and saturation was 98% with 6 L oxygen. Her right leg was pale and painful, without arterial pulsation. She enabled to bend the right knee and the right ankle in a dorsal direction. Doppler ultrasound detected a faint pulse in her right femoral artery but could not detect it in the right popliteal artery. Her left leg presented a mild edematous change. Enhanced computed tomography (CT) revealed occlusion of the right common and external iliac arteries (**Fig. 1A**). The bilateral peripheral arteries below the knee had multiple stenoses. A deep venous thrombus was observed in the left popliteal vein (**Fig. 1B**). Additionally, a tumor in the right main bronchus was revealed, which occluded the entry of both the right middle and lower lobar bronchi (**Figs. 1C** and **1D**). A mass observed to extend into the left atrium (LA) was considered to be the invasion of the tumor (**Fig. 1E**). The mediastinal lymph nodes were swollen. The bilateral kidneys had plural small infarcted lesions, and the left adrenal gland was swollen with a low-density tumor (**Fig. 1F**). The mass in the LA was examined using echocardiography, and it had high mobility (**Fig. 2A**) and continuity with the tumor in the right main bronchus (**Fig. 2B**). The tumor was considered to be primary lung cancer with invasion of the LA, and metastasis to the bilateral kidneys and left adrenal gland was suspected. Nevertheless, it was dangerous to make a diagnosis using direct biopsy because of the highly mobile tumor in the LA.

**Figure 3:**
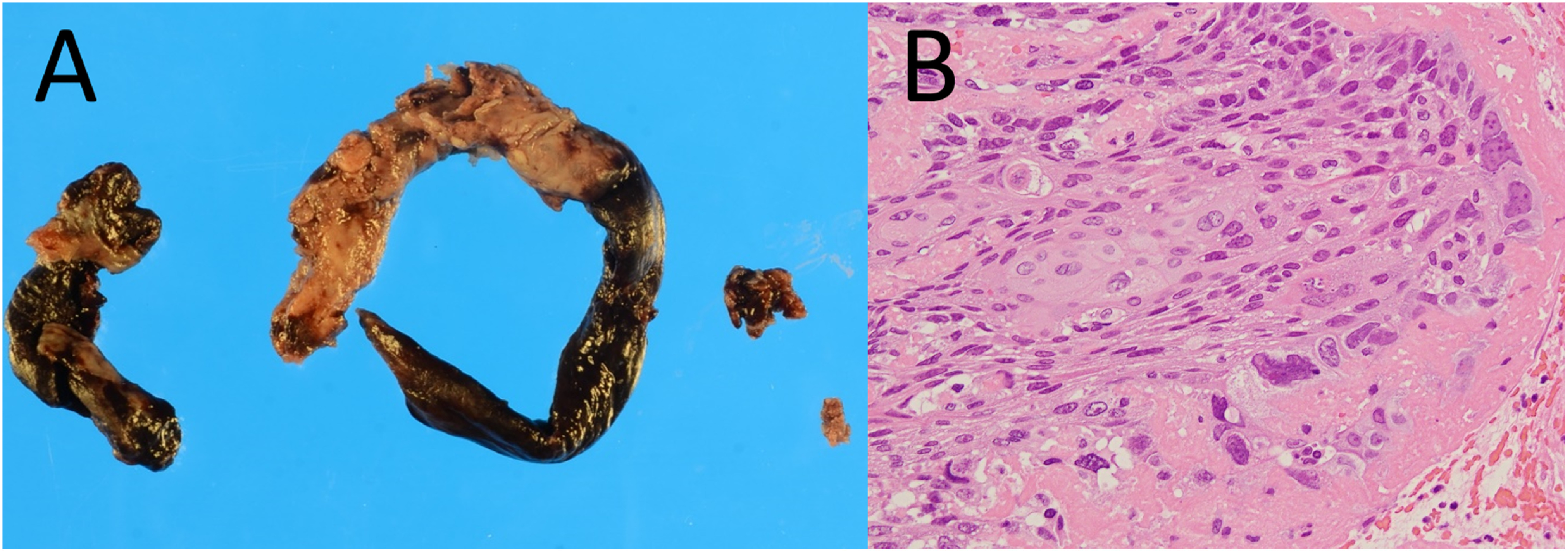
Fig. 3 Findings of the embolus. (**A**) Picture of the embolus obtained from right common and external iliac arteries. It was brown and solid, mixed with a clot. (**B**) Pathological finding of the embolus. Polygon cancer cells presented sheet-like proliferation, which led to the diagnosis as squamous cell carcinoma.

The right lower extremity required emergency surgical intervention to avoid limb amputation. Embolectomy of the right iliac artery was performed via the right common femoral artery (CFA). No emboli were observed in the CFA. Using the Fogarty catheter, the embolus in the common and external iliac arteries was removed. It was solid with a light brown color and not only a clot (**Fig. 3A**). Following confirmation of antegrade and retrograde blood flow, angioplasty of the CFA was performed. After the surgery, the pulses of the right anterior and posterior tibial arteries were perceived using Doppler ultrasound. The ischemic time of the patient’s right limb was estimated to be approximately 6.5 h. The patient’s postoperative course was uneventful. Enhanced CT showed the patent iliac artery. The embolus obtained by embolectomy was elucidated through microscopic examination, which resulted in squamous cell carcinoma mixed with blood components (**Fig. 3B**). Postoperative brain magnetic resonance imaging showed microembolisms in the bilateral cerebral hemispheres and left hemispherium cerebelli.

The patient was diagnosed with primary lung cancer of stage IV squamous cell carcinoma (cT4N2M1). The effective dose of chemotherapy or radiotherapy was assessed to be difficult to achieve because of her general condition. Furthermore, the adverse event of antitumor effects, such as scattering embolus, was a concern. Radiotherapy also poses a risk of myocardial damage. The patient ultimately decided not to undergo aggressive treatment and was admitted to a hospice. She was discharged 1 month after surgery on her foot.

## Discussion

Tumor embolism caused by primary lung cancer is extremely rare. Miroslav et al. reported a low prevalence of malignancy-related embolism, and only 0.3% of cancer patients presented with arterial embolism,^1)^ which rarely occurred spontaneously.^2,3)^ The majority of malignancy-related arterial embolism cases were reported to be associated with lung cancer.^4)^ In the present case, specifying the cause of acute limb ischemia with severe symptoms was difficult. Initially, an enhanced CT scan was performed to identify pulmonary embolism; however, it revealed the primary lung cancer invading the LA. Some studies have reported that advanced lung cancer should be considered to be a differential diagnosis of arterial embolism.^3,5–7)^ The present case had a highly mobile mass in the LA, which was responsible for acute lower extremity ischemia. Since the mass could result in thrombus formation in the LA, we conducted echocardiography to confirm that it originated from the primary lung cancer with continuity. Additionally, the method for making a diagnosis of lung cancer was carefully considered because of the risk of scattering the fragments during biopsy. Thus, we performed an embolectomy to make a diagnosis combined with limb salvage and ultimately confirmed the squamous cell carcinoma on the basis of pathological findings.

Malignancy-related hypercoagulability (Trousseau’s syndrome) is a differential diagnosis of infarction. The patient’s brain and kidneys had multiple small infarcts, which were not definitively diagnosed as either tumor-fragment embolism or Trousseau’s syndrome. She also had a thrombus in the left popliteal vein, which was considered a symptom of Trousseau’s syndrome. Kanaji et al. reported that 2.2% of patients with lung cancer have thromboembolism, and patients with thromboembolism have a shorter overall survival period than patients without thromboembolism.^8)^ Rigdon also reported that the prognosis for limb salvage and long-term survival of patients with Trousseau’s syndrome is poor.^9)^ In the present case, the pathophysiology of lower limb ischemia was considered to be tumor-scattering embolism and thrombosis caused by malignancy-related hypercoagulability.

We performed an embolectomy for the ischemic limb, which contributed to maintaining the patient’s quality of life in the terminal phase. Togo et al. reported that the acute ischemic limb that develops because of primary lung cancer may require limb amputation.^5)^ Loscertales et al. also reported surgical intervention to remove a tumor in the LA that was floating without atrial wall invasion in a patient with pulmonary adenocarcinoma; however, the patient died postoperatively.^10)^ The success of the surgical intervention might depend on the degree of adhesion with surrounding tissues, including the wall of the LA. We decided not to perform surgery to excise the primary lesion for two reasons: (1) there was a possibility of strong adhesion with the LA that could have resulted in the incomplete removal of the cancer and (2) the patient’s general condition was not stable enough to endure an extended operation. The prognosis was reported to be highly correlated with the TNM stage.^5)^ Nonetheless, even if a patient has an advanced stage of cancer, it is beneficial to excise the embolus to make a diagnosis and salvage the limb.

## Conclusion

We encountered a case of primary lung cancer that invaded the LA and scattered into the right iliac artery, which developed an acute ischemic limb. Malignancy-related hypercoagulability was also existed as the deep venous thrombus in the left leg. A pathological diagnosis was made using the fragment obtained via embolectomy. Advanced lung cancer should be considered as a differential diagnosis for acute ischemic limbs. Successful limb rescue contributed to maintaining the patient’s quality of life, even in the terminal phase.
